# The Effect of Treatment History on Therapeutic Outcome: Psychological and Neurobiological Underpinnings

**DOI:** 10.1371/journal.pone.0109014

**Published:** 2014-10-02

**Authors:** Simon Kessner, Katarina Forkmann, Christoph Ritter, Katja Wiech, Markus Ploner, Ulrike Bingel

**Affiliations:** 1 Department of Neurology, University Medical Center Hamburg-Eppendorf, Hamburg, Germany; 2 Department of Neurology, University Hospital Essen, University Duisburg-Essen, Essen, Germany; 3 Nuffield Department of Clinical Neurosciences, John Radcliffe Hospital, University of Oxford, Oxford, United Kingdom; 4 Oxford Centre for Functional Magnetic Resonance Imaging of the Brain, Nuffield Department of Clinical Neurosciences, John Radcliffe Hospital, University of Oxford, Oxford, United Kingdom; 5 Department of Neurology, Technische Universität München, Munich, Germany; Université catholique de Louvain, Belgium

## Abstract

It is increasingly recognized that the efficacy of medical treatments is determined in critical part by the therapeutic context in which it is delivered. An important characteristic of that context is treatment history. We recently reported first evidence for a carry-over of treatment experience to subsequent treatment response across different treatment approaches. Here we expand on these findings by exploring the psychological and neurobiological underpinnings of the effect of treatment experience on future treatment response in an experimental model of placebo analgesia with a conditioning procedure. In a combined behavioral and neuroimaging study we experimentally induced positive or negative experiences with an analgesic treatment in two groups of healthy human subjects. Subsequently we compared responses to a second, different analgesic treatment between both groups. We found that participants with an experimentally induced negative experience with the first treatment showed a substantially reduced response to a second analgesic treatment. Intriguingly, several psychological trait variables including anxiety, depression and locus of control modulate the susceptibility for the effects of prior treatment experiences on future treatment outcome. These behavioral effects were supported by neuroimaging data which showed significant differences in brain regions encoding pain and analgesia between groups. These differences in activation patterns were present not only during the pain phase, but also already prior to painful stimulation and scaled with the individual treatment response. Our data provide behavioral and neurobiological evidence showing that the influence of treatment history transfers over time and over therapeutic approaches. Our experimental findings emphasize the careful consideration of treatment history and a strictly systematic treatment approach to avoid negative carry-over effects.

## Introduction

Experimental and clinical observations suggest that treatment outcome is not solely determined by the genuine characteristics of a treatment (e.g., pharmacological properties), but also depends in critical part on the therapeutic context in which it is delivered [1]. A key element of treatment context is the prior experience with a treatment that is known to modulate treatment efficacy [2], [3]. The pivotal role of this factor for the therapeutic outcome is most apparent in experimental placebo analgesia studies involving a conditioning component [4]–[7]. In these studies the administration of a placebo treatment is combined with a conditioning procedure (e.g., reduction of pain stimulus intensity) in which participants experience analgesia and thereby learn the efficacy of the treatment. Subsequent responses to the same placebo treatment are substantially enhanced after this positive learning experience. Importantly, the impact of prior experience on treatment outcome is not limited to placebo (i.e., inert) treatments. Prior experience also modulates the efficacy of a subsequently applied active treatment, presumably by inducing treatment-specific expectations, via (pharmacological) conditioning or a combination of both mechanisms; for review see [3]. To date, this influence of prior treatment experience has only been studied *within the same treatment* approach. However, in clinical practice treatments are commonly replaced by another approach (e.g., a drug with a different pharmacological profile), particularly if symptoms show no or insufficient improvement. Whether the experience with one treatment approach can ‘carry over’ to a subsequent, different treatment, is currently unknown.

To allow for the assessment of treatment history effects irrespective of pharmacological peculiarities, analgesia was modeled experimentally by using an analgesic placebo paradigm. Positive or negative experience with an analgesic patch treatment was experimentally induced using a conditioning procedure [8]. On day 3, the analgesic effect of a second placebo treatment introduced as an ointment with an unrelated pharmacological profile was compared between the positive and the negative treatment history group. Functional magnetic resonance imaging (fMRI) was performed as a physiological measure of analgesia and to characterize the brain mechanisms underlying the influence of treatment history on treatment outcome. We hypothesized that the individuals' experience with the first treatment carries over to the next treatment approach and thereby modulates subjective as well as objective indicators of subsequent treatment efficacy.

As previously reported in a short research letter [9], we found that the therapeutic effect of the second treatment approach (indexed by pain intensity ratings) was significantly higher in the positive compared to the negative treatment history group. This behavioral finding was corroborated by fMRI data indicating smaller treatment induced changes in posterior insula activity in the negative compared to the positive group. Additionally, the positive group showed stronger activity in the right dorsolateral prefrontal cortex during painful stimulation.

Here we expand on these findings by further exploring the psychological and neurobiological underpinnings of the effect of treatment experience on future treatment response. We hypothesized that individual psychological variables such as anxiety and depressiveness may influence the effects of the treatment itself and/or treatment history. Further we aimed at exploring the relationship between pain regulatory responses during pain anticipation and the effects of treatment history on pain related activity during thermal stimulation [10]. Finally, we provide a detailed discussion of all data, their potential implications for clinical practice and scientific questions to be addressed in future studies.

## Methods

### Participants

Forty healthy volunteers (all right handed, mean age: 26 years; range: 22 to 36 years, 20 male) participated in the study and were randomly assigned to either the positive or negative treatment experience group. The groups did not differ significantly with respect to age and gender. All participants had normal heat pain thresholds on their left forearm where painful stimuli were applied [11]. None of them was taking any medication except oral contraceptives. Participants had no known history of neurological or psychiatric diseases, including recurrent or chronic pain. The study was conducted in accordance with the Declaration of Helsinki and had been approved by the local ethics committee (Ärztekammer Hamburg, Ethik-Kommission, Hamburg, Germany). All participants gave written informed consent to participate and were free to withdraw from the study at any time. After the end of the study, participants were debriefed about the actual aim of the study and that only placebo treatments had been used. Due to technical failure of the thermode, data of one participant had to be discarded. The final data analysis is therefore based on 39 subjects (19 in the positive group).

### Experimental procedures

**Figure 1:**
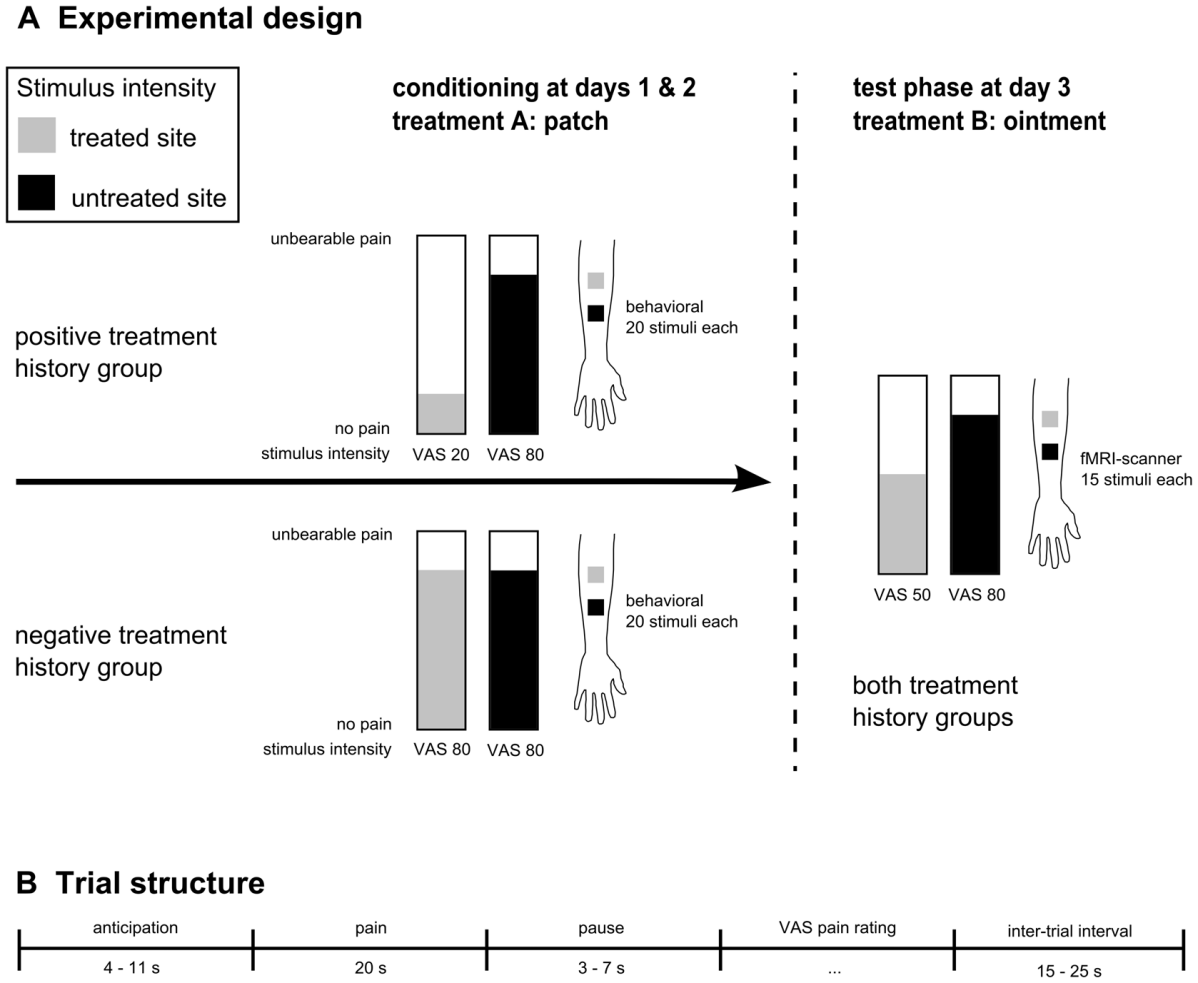
The experimental design comprised a group-specific conditioning phase with treatment A and a test phase using treatment B (A). The experiment took place on three consecutive days. On days one and two either a positive or negative treatment experience was induced by combining an inert patch treatment with a conditioning procedure. On day three the analgesic response to a second analgesic treatment applied as an ointment was assessed. Bars indicate the stimulation intensities of applied heat pain stimuli for the conditioning and the test session. In the conditioning session on days one and two (left-hand side) an inert patch (treatment A) was attached to the left forearm and after a waiting period of 20 minutes a series of 20 heat pain stimuli was applied to the untreated (black) and the treated site (gray) in randomized order. In the positive treatment history group, a low stimulus intensity (VAS 20) was applied to the patch treated site to mimic analgesia while an intensity of VAS 80 was applied to the untreated site. In the negative treatment history group the same stimulation intensity of VAS 80 was applied to the untreated and the treated site. The test phase performed with fMRI on day three was identical for both groups. Participants were instructed that another analgesic with a different pharmacological profile would be administered and an inert white ointment was applied to the participants' left forearm (treatment B). After a waiting period of 20 min two series of 15 painful heat stimuli were applied to the treated site and the untreated site in a randomized order. In both groups, a stimulus intensity of VAS 80 was applied to the untreated site and a stimulus intensity of VAS 50 was applied to treated site to mimic the analgesic effect of treatment B. **Trial structure of each pain stimulus (B).** Each trial consisted of three phases: anticipation, pain, and rating. The anticipation phase began when the white crosshair that was displayed on the computer screen turned into a red crosshair, indicating that a painful stimulation would follow shortly. Subjects had to press a button as quickly as possible when the crosshair changed color. After a variable delay a 20 s painful thermal stimulus was administered. Three to seven seconds after the thermal stimulation, subjects had to rate the pain intensity using a VAS. The trial was completed by a 15–25 seconds baseline during which a white crosshair was displayed.

The experiment took place on three consecutive days. On days 1 and 2, either a positive or a negative treatment experience was induced by combining an inert patch treatment with a *conditioning procedure* [8]. During the *test phase* performed on day 3 the analgesic response to a second analgesic treatment applied as an ointment was assessed behaviorally and using fMRI, see Fig. 1 A.

### Conditioning phase (days 1 and 2)

Participants were recruited with the understanding that the purpose of the study was to investigate brain mechanisms responsible for inter-individual differences in the efficacy of analgesics. Upon arrival for the first session, subjects were informed about the experimental procedures and written informed consent was obtained. All instructions were read to the participants to ensure a standardized procedure. To assess participants' anxiety, depression and locus of control, participants filled in the State Trait Anxiety Inventory [12], [13], the Center for Epidemiologic Studies Depression Scale [14]; German version: ADS-K [15], and the IPC questionnaire about locus of control [16], [17]; German version: [18].

For the noxious stimulation, a contact heat stimulus delivery thermode (30×30 mm^2^, ATS-Thermode, Pathway System, Medoc) was attached to the middle inner aspect of the left forearm. First, the individual heat pain threshold at the site of stimulus application was determined using the Method of Limits [19]. Subsequently, participants were familiarized with the equipment and pain rating procedure by presenting several stimuli of different temperature levels to the left forearm. The participants were asked to rate each pain stimulus on a Visual Analog Scale (VAS, [100 parts; endpoints labeled as “no pain” and “unbearable pain”]). The VAS was displayed on a computer screen using the experimental control program Presentation (www.neurobs.com). In the MR scanner the VAS was displayed via a video projector and a mirror on the head coil. Outside the scanner, subjects used computer mouse buttons to rate each pain stimulus. During the fMRI scan, a button box was used instead of a computer mouse. Second, a temperature calibration was performed to determine the individual temperatures to evoke pain levels of 20, 50 and 80 on the VAS. To this end, a pseudo-randomized sequence of 16 thermal stimuli (duration: 20 seconds) of different temperature levels was applied. Participants were instructed to rate the intensity of each stimulus on the VAS. Based on these ratings, stimulation intensities corresponding to subjective pain intensity ratings of VAS 20, 50 and 80 were determined by means of regression analysis to ensure a perception-locked stimulation across subjects.

Next, negative or positive treatment experience was induced by a conditioning procedure. To this end, an inert skin patch (treatment A) was attached to the left forearm. The 7×5 cm^2^ patch consisted of a white cotton tissue with an inert sticky layer that was attached to the skin. Participants were informed that after a short period during which the drug would be absorbed the analgesic effect of the drug was expected to last for at least one hour. After 20 minutes the patch was removed. A rectangle was drawn around the treated site after the patch had been removed. This ensured that the subjects knew whether the treated or the untreated site would be stimulated next. Furthermore, participants were verbally instructed which site would be stimulated in the next block. During the actual conditioning session, a series of each 20 heat pain stimuli (duration 20s, ITI 40s) was applied to the treated or an untreated site in randomized order. The intensity of the stimuli on the treated site differed between subjects depending on the assignment to the ‘negative’ or the ‘positive’ treatment history group. In the positive treatment history group, a low stimulus intensity (VAS 20) was applied to the patch treated site to mimic analgesia while an intensity of VAS 80 was applied to the untreated site. This manipulation was unbeknownst to the participants. In contrast, the negative treatment history group received the same stimulation intensity of VAS 80 on the untreated and the treated site. In order to reinforce the positive or negative drug experience with the patch this conditioning procedure was performed twice and thus repeated on two consecutive days in both groups.

Each trial consisted of three phases: anticipation, pain, and rating. The anticipation phase began when the white crosshair that was displayed on the computer screen turned into a red crosshair, indicating that a painful stimulation would follow shortly. Subjects had to press a button as quickly as possible when the crosshair changed color. After a variable delay (7.5s±3.5), a 20s painful thermal stimulus was administered (1.5s ramp up, 17s plateau, 1.5s ramp down). A variable delay (5s±2) followed the thermal stimulation before subjects had to rate the level of pain during the trial using a VAS. A variable inter-trial interval (ITI; 20s±5) followed during which a white crosshair was displayed (see Fig. 1 B).

### Test phase (day 3)

The test phase was identical for both groups. On day 3, both groups were instructed that another analgesic with a different pharmacological profile would be administered. Subsequently, an inert white ointment was applied to the participants' left forearm (treatment B). Subjects were told that the cream contained a different analgesic agent than the one that had been applied on days 1 and 2. In order to emphasize the difference between the two ‘drugs’, an ointment was chosen rather than a patch. The ointment was removed 20 min after administration. Thereafter, subjects were positioned in the MRI scanner and two series of 15 painful heat stimuli each were applied to the treated site and the untreated site. The order was randomized across subjects, i.e., in half of the participants the untreated site was stimulated first, while in the other half the treatment site was stimulated first. As in the conditioning session, a stimulus intensity of VAS 80 was applied to the untreated site. In both groups, a stimulus intensity of VAS 50 was applied at the ointment treated site. This procedure was chosen to mimic the effects of a novel analgesic treatment. The analgesic effect of this second treatment (as indexed by pain ratings) was compared between the positive and the negative treatment history group. In addition, functional magnetic resonance imaging (fMRI) was performed to assess pain-related brain activity as a physiological measure of analgesia. Importantly, both groups underwent exactly the same procedure of treatment with the ointment (treatment B). The only difference between the groups was their different prior experience with treatment A.

### FMRI data acquisition

MR scanning during the test phase was performed on a 3 Tesla system (TimTrio, Siemens) equipped with a 32-channel head coil. A total of 42 axial slices per volume were acquired in top-down order using a gradient-echo echo-planar-imaging (EPI) T2*-sensitive sequence with the following parameters: TR = 2580 ms; TE = 26 ms; voxel size = 2×2×2 mm^3^; gap between slices = 1 mm; flip angle = 90°; field of view = 220×220 mm^2^; time of acquisition = 30 minutes. After the functional measurement, an individual high-resolution anatomical image was obtained for each participant using a T1-weighted magnetization-prepared rapid acquisition gradient-echo (MPRAGE) sequence (TR = 2300 ms; TE = 2.98 ms; voxel size = 1×1×1 mm^3^; flip angle = 9°; field of view = 256×256 mm^2^; time of acquisition = 7.23 minutes).

### Analysis of behavioral data

Behavioral data (e.g. pain ratings, reaction times etc.) were automatically recorded and logged by the experimental control program Presentation. All behavioral data analyses were conducted using SPSS 18.0.

Behaviorally, the individual analgesic effect of the second analgesic treatment was defined as the difference between the pain ratings for the untreated site and the treated site. The mean analgesic effect was compared between treatment history groups using two-sample T-Tests. Pearson correlation coefficients were calculated to examine the relationship between the different experimental effects and questionnaire data.

Results with p-values<0.05 are considered statistically significant. All statistical analyses were performed using two-tailed testing. Results are presented as mean ± standard error unless indicated otherwise.

### Image processing and statistical analysis

Image processing and statistical analysis of fMRI data was performed using SPM8 (http://www.fil.ion.ucl.ac.uk/spm/) [20], [21]. After removing the first 6 volumes to compensate for T1 saturation effects, preprocessing included slice timing, realignment to the first volume, unwarping (correction for interaction of movements and field inhomogeneities), normalization to standard MNI space and finally smoothing with an 8 mm Gaussian kernel with full-width at half-maximum (FWHM). Data were also subjected to high-pass filtering (cut-off period: 128 s) and correction for temporal autocorrelations (based on a first-order autoregressive model).

Data analysis was performed using a general linear model approach. For each subject the first level design matrix included 2 experimental runs (i.e., stimulation of the treated and the untreated site) each of which had 4 regressors that modelled the predicted blood oxygen level dependent (BOLD) responses to the different events that comprised one pain trial. The painful stimulation was divided into an early and a late phase based on previous results regarding neural placebo effects [6], [10]. First level models therefore comprised the following regressors: (i) the pain anticipation phase, (ii) the early phase of painful thermal stimulation, (iii) the late phase of painful stimulation and (iv) the pain rating procedure. All regressors were obtained by convolving the duration of the event with the canonical hemodynamic response function implemented in SPM.

After model estimation, the ensuing first-level contrast images from each subject were used for second-level analyses applying SPM's flexible factorial model that comprised the factors group (negative vs. positive), condition (untreated vs. treated site) and the individual subject factor. Inferences were made by entering the appropriate contrast into an ANOVA using a flexible factorial design and correcting for possible non-sphericity of the error term.

Our analysis comprised five steps. First, we tested whether the noxious stimulation had activated brain regions implicated in pain processing and perception (main effect of pain). For this analysis, pain-related responses were pooled across all four conditions of the 2×2 factorial design.

Next, we tested for differences in pain-related responses depending on the stimulation site (main effect of treatment). Given that the analgesic treatment effect was mimicked by applying a reduced stimulus intensity corresponding to VAS 50 on the treated site compared to the untreated site (VAS 80), we expected activity changes in brain areas known to code pain intensity in this contrast.

Third, we tested for the main effect of treatment history by comparing pain related responses between the negative and the positive group.

Forth, we tested whether the difference in pain-related responses between the treated and the untreated site differed depending on treatment history group (interaction).

Finally, we tested for group-specific activity patterns and the relationship between pain regulatory responses during pain anticipation and the reduction of pain related activity during thermal stimulation. To this end, the difference in parameter estimates for the untreated and treated condition during painful stimulation was extracted from a 4 mm radius sphere centered on the insular peak voxel (as a neurobiological marker for the individual treatment effect) and used as a covariate for brain responses during the anticipation phase.

We report p-values at a level of p≤0.05 which have been corrected for multiple comparisons, as obtained both from whole brain correction (marked with two asterisks [**]) and from small volume correction (one asterisk [*]) from a-priori regions of interest. Correction was based on spheres centred around the peak coordinates (ignoring laterality) obtained from previous studies [10], [22]–[25] or using an anatomical mask [26], [27] in case of the dorsolateral prefrontal cortex. These regions include brain areas known to be involved in pain processing (brainstem (3/−35/−17), thalamus (12/−12/4), basal ganglia (8/14/−10), posterior (48/−4/16) and anteromedial (34/0/−6) insula and secondary somatosensory cortex (49/−19/18) [28]) and its modulation (subgenual (10/10/−10) and dorsal (8/34/18) anterior cingulate cortex, dorsolateral prefrontal cortex (Brodmann area 9 [29]), amygdala (16/−6/−15; 24/−2/−18) and hippocampus (21/−21/−9) [30], [31]). Activation in cortical areas was corrected using 15 mm radius spheres, subcortical areas were corrected using 10 mm radius spheres. For exploratory purposes we also report uncorrected results on a more lenient threshold of p≤0.001 [**^†^**].

## Results

Note that part of the behavioral data have been published elsewhere [9].

### Behavioral data

All participants had normal thresholds to heat pain at the site of stimulus application (volar forearm, 45.8°C±2.7). The mean temperatures for the test stimuli that corresponded to a pain sensation of 20, 50 and 80 on the VAS as determined in the calibration session were 44.1°C±1.3, 45.9°C±1.1 and 46.9°C±0.9, respectively.

**Figure 2:**
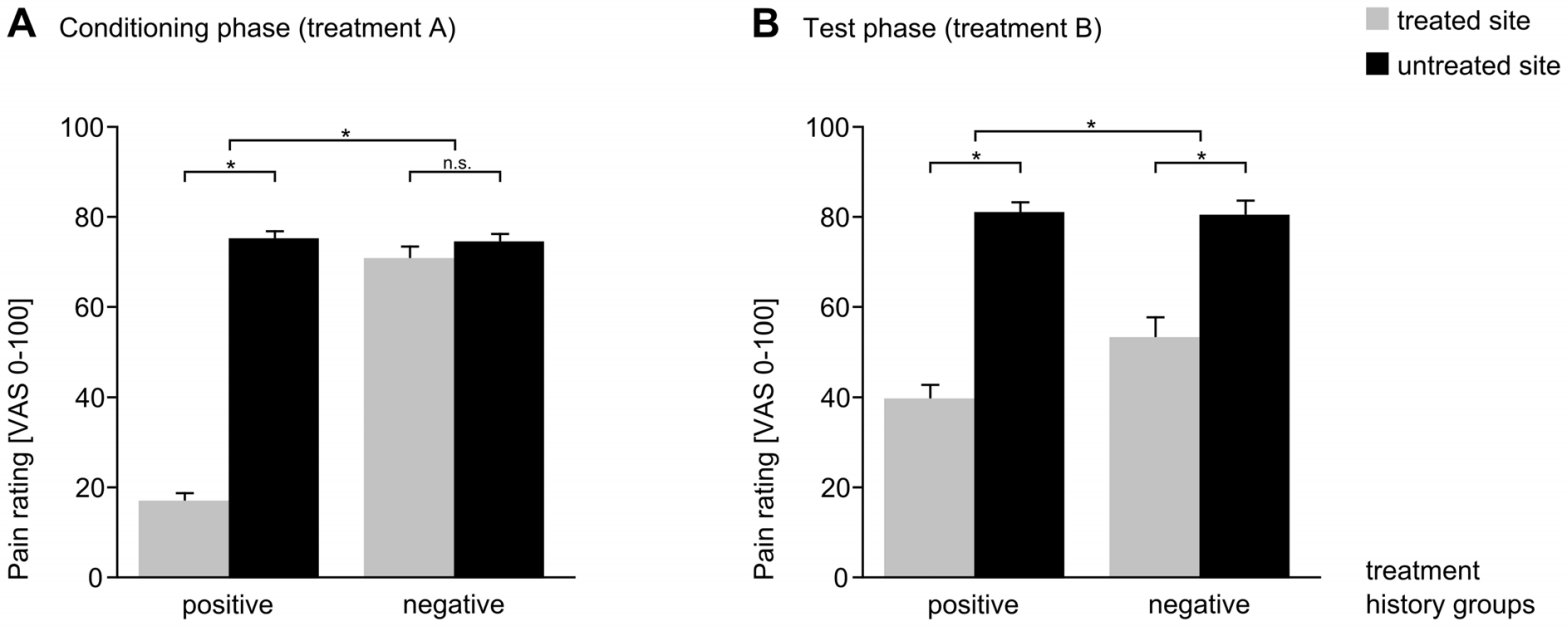
Behavioral effects during conditioning and test phase. Treatment experience during the conditioning session (**A**). Pain ratings (mean VAS score ± SEM) indicate a successful manipulation of the treatment experience for both groups with the placebo patch (treatment A). The effect of treatment history on treatment outcome - response to second treatment (**B**). Pain ratings indicate that the therapeutic effect (mean pain VAS score ± SEM) of the ointment treatment (treatment B) was significantly lower in the negative than in the positive treatment history group. See the difference between groups in gray bars. Error bars indicate standard error of the mean. * = p<0.01.

Mean pain ratings during the *conditioning phase* differed between groups. Pain ratings on the VAS were 17.3±1.9 on the treated site and 75.5±1.8 on the untreated site in the positive group and 71.1±2.8 on the treated site and 74.8±1.9 on the untreated site in the negative group. Pain relief from the placebo patch (treatment A) was significantly greater in the positive group (Δ VAS = 58.2) compared to the negative group (Δ VAS = 3.7, t_37_ = −13.9, p<0.001), indicating a successful manipulation of treatment experience (Fig. 2 A).

In the *test phase*, the analgesic effect of the second, different treatment was compared between the positive and the negative treatment history group. This analysis revealed that the therapeutic effect of the ointment (treatment B) was significantly greater in the positive treatment history group than in the negative group (positive group: Δ VAS = 41.3, negative group: Δ VAS = 27.1, t_37_ = −2.9, p = 0.007, (Fig. 2 B). Positive group: VAS 40.0±3.3 on the treated site and VAS 81.3±2.4 on the untreated site, negative group: VAS 53.6±4.6 on the treated site and VAS 80.7±3.4 on the untreated site [9]). Note, that physically identical stimulation temperatures were used in both groups (mean applied temperature: 45.9°C±0.2 at the treated and 46.9°C±0.1 at the untreated site).

Across all participants, the analgesic benefit from the second analgesic treatment was negatively correlated with individual trait anxiety (r = −0.4, p<0.05).

Within the positive group, a negative correlation between depression score (ADS-K) and treatment response was observed (r = −0.53, p<0.05). Furthermore, in the negative group, a correlation analysis of the psychological characteristic ‘locus of control’ and the individual treatment response showed that subjects with a more external locus of control benefited even less from the analgesic treatment (r = −0.68, p<0.05).

### FMRI

**Figure 3:**
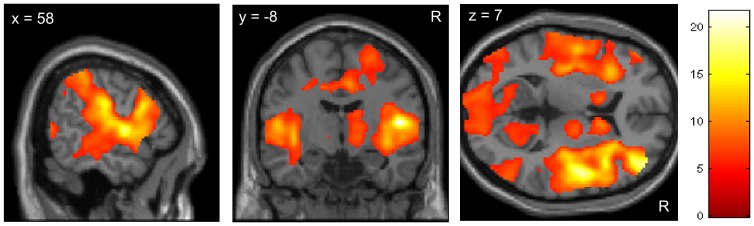
Pain-related BOLD response (main effect of pain). The 20 s thermal stimulus induced significant activation (t-score) of pain-processing regions including the thalamus, striatum, insula, S I, S II, MCC and prefrontal cortices. The image is thresholded at p<0.05 FWE whole brain correction. For a complete list of activated brain areas see Table 1 A.

We first identified brain areas which responded to the painful thermal stimulation, pooled across all four experimental conditions. The results show that the painful stimuli significantly activated the well-known cerebral pain network [28], including the primary and secondary somatosensory cortices (S I, S II), the insula and the midcingulate cortex (MCC). Subcortical responses were recorded in the thalamus, basal ganglia, brainstem and cerebellum (Table 1 A and Fig. 3).

**Table 1:** FMRI results.

Region	MNI-coordinates in mm						Voxel level (T)
	right			left			right/left
	X	Y	Z	X	Y	Z	
***(A) Main effect of painful stimulation (pooled across all four experimental conditions)***							
Posterior insula	50	−8	10	−52	−8	8	21.6**/12.1**
S II	36	−18	20	−36	−20	14	21.2**/15.9**
DLPFC	46	48	12	−46	32	22	21.1**/8.2**
Middle insula	56	0	4	−54	−4	4	18.7**/11.2**
Anterior insula	44	16	6	−36	22	6	16.2**/12.6**
SMA	6	18	52				15.6**
Cerebellum	30	−64	−44	−34	−60	−44	7.7**/12.8**
MCC	8	−8	42	−10	24	38	10.0**/9.8**
Head of caudate	10	12	10	−10	12	8	9.6**/8.4**
Occipital lobe	10	−92	2	−8	−92	−6	8.3**/9.3**
Thalamus	16	−10	6	−10	−6	0	8.2**/5.6**
PAG	10	−30	−8				8.1**
S I	30	−36	64				7.8**
Amygdala	32	−2	−16	−34	−2	−22	7.7**/5.5**
***(B) Main effect of condition [untreated site>treated site]***							
Amygdala				−16	−2	−8	3.4*
Insula	40	8	2	−44	6	2	3.0†/3.4*
MCC	2	8	42				3.1†
***(C) Main effect of group [Negative>Positive group]***							
Medial frontal gyrus	50	44	20				5.9**
Occipital pole	2	−94	16	−16	−88	−4	5.3†5.1†
Superior parietal lobe	16	−66	64				4.4†
Amygdala	8	−4	−20	−8	−4	−20	4.1*/3.9*
S II	50	−16	16				3.5*
***(D 1) Interaction in complete pain phase [Positive group _[untreated>treated site]_>Negative group _[untreated>treated site]_]***							
Insular cortex	48	−8	10	−40	−8	−4	4.0*/3.6*
***(D 2) Interaction in late pain phase [Positive group _[untreated>treated site]_>Negative group _[untreated>treated site]_]***							
Insular cortex	48	−6	8	−46	−10	0	3.6*/3.1†
***(E 1) Interaction in complete pain phase [Positive group _[treated>untreated site]_>Negative group _[treated>untreated site]_]***							
Amygdala				−22	−2	−26	3.7*
Head of caudate				−18	10	−16	3.6*
Subgenual ACC				−10	8	−12	3.4*
Orbitofrontal cortex	22	14	−22	38	44	−20	3.2†/2.9†
***(E 2) Interaction in late pain phase [Positive group _[treated>untreated site]_>Negative group _[treated>untreated site]_]***							
Striatum/subgenual ACC				−12	6	−14	3.5*
DLPFC	42	38	40				3.2*
Amygdala	32	4	−30				3.2†
Orbitofrontal cortex	20	16	−20				2.8†
***(F 1) Negative group during anticipation phase [T-test: negative correlation with treatment effect during pain]***							
Insular cortex	44	−6	−4	−44	−2	−4	4.3*/5.5*
Thalamus	20	−6	2	−20	0	4	4.9*/4.2†
Cerebellum	6	−42	−14	−4	−42	−12	3.7†/4.5†
***(F 2) Positive group during anticipation phase [T-test: positive correlation with treatment effect during pain]***							
PAG	2	−20	−16	−2	−20	−16	5.4*/4.0*
Medial frontal gyrus				−8	34	48	5.2†
Cerebellum	32	−82	−26				5.1†
DLPFC	36	34	42	−32	26	44	4.1*/4.2*
dACC	6	36	8	−8	42	18	3.7†/4.0*
Midbrain	16	−10	−10	16	−10	−10	3.7†

Areas of brain activation (BOLD responses from fMRI) during test phase (treatment B).

S I = primary somatosensory cortex, S II = secondary somatosensory cortex, DLPFC = dorsolateral prefrontal cortex, SMA = supplementary motor area, MCC = middle cingulate cortex, PAG = periaqueductal gray, dACC = dorsal anterior cingulate cortex;

(**) p<0.05 whole brain corrected,

(*) p<0.05 small volume corrected using respective a-priori region of interest,

(^†^) p<0.001 uncorrected.

Comparing pain-related responses to stimulation on the untreated and the treated site (main effect of treatment) across both treatment history groups revealed stronger activity in the bilateral insular cortex, MCC and amygdala for the untreated compared to the treated site, reflecting the higher stimulation intensity on this site during the test phase (Table 1 B).

The main effect of group revealed stronger pain-related activity in the amygdala, S II and frontal cortex in the negative compared to the positive group. Furthermore, this group showed stronger activity in the occipital and superior parietal lobes (Table 1 C).

**Figure 4:**
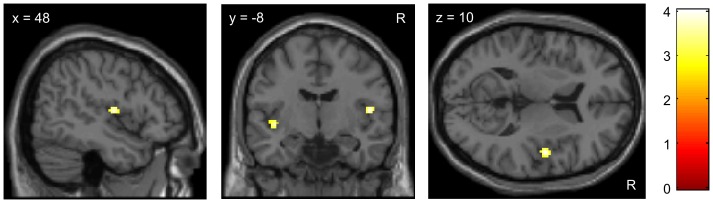
The effect of treatment history on analgesic outcome is reflected in pain-related responses in the posterior insula. FMRI revealed less reduction of pain-related BOLD activity (t-scores) on the treated compared to the untreated site following a negative treatment history compared to a positive treatment history. Activation related to the ‘group-by-condition-interaction’ contrast *[Positive group [untreated>treated site]>Negative group [untreated>treated site]],* for details see Table 1 D1. For visualization purposes the images are thresholded at p<0.005.

We then tested whether BOLD responses to pain reflect the behavioral differences in analgesia to the second treatment. We expected less reduction of pain-related activity on the treated compared to the untreated site in the negative treatment history group compared to the positive group. Indeed, the interaction contrast [Positive group _[untreated>treated site]_>Negative group _[untreated>treated site]_] revealed bilateral activity in the posterior insular cortex, which was stronger at the right side, corresponding to painful stimulation performed on the left forearm (Table 1 D1 and Fig. 4), as shown in [9]. This response pattern was evident during both pain phases (early and late).

**Figure 5:**
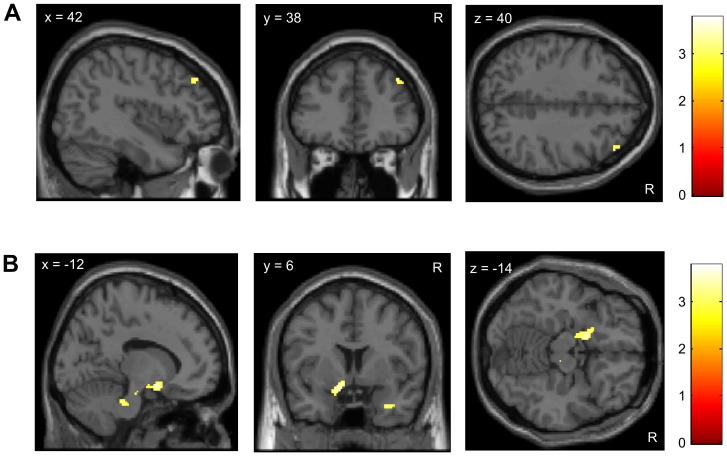
Increased analgesic response to new treatment following a positive treatment history is associated with higher pain related activity in the right dorsolateral prefrontal cortex (DLPFC, A) and the striatum (B). Images show BOLD responses (t-scores) to painful heat stimulation related to the ‘group-by-condition-interaction’ contrast *[Positive group [treated>untreated site]>Negative group [treated>untreated site]]*, for details see Table 1 E2. For visualization purposes the images are thresholded at p<0.005.

Furthermore, we aimed to identify brain regions that mediated the increased analgesic response to the second treatment in the positive compared to the negative treatment history group. We therefore investigated the opposite ‘group-by-condition-interaction’ contrast [Positive group _[treated>untreated site]_>Negative group _[treated>untreated site]_] and expected that brain areas involved in the descending modulation of pain mediated the analgesic benefit from treatment B in the positive treatment history group. Indeed, we found that the stronger analgesic effect in the positive compared to the negative treatment history group was associated with activity within the striatum and anterior cingulate cortex (ACC) during both pain phases and in the right dorsolateral prefrontal cortex (DLPFC) during the late pain phase [9]. These areas are key regions of descending pain modulation (Table 1 E1, E2 and Fig. 5 B).

**Figure 6:**
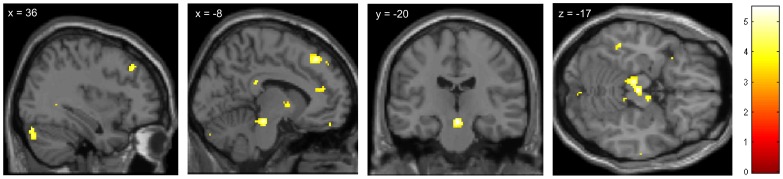
Increased treatment response in the positive group is associated with increased activity of pain regulatory networks during the anticipation of pain. Images show BOLD activity (t-scores) during the anticipation phase (t-test: positive correlation of anticipatory activity *[treated>untreated site]* with the treatment response in the posterior insula for the positive group). Stronger reductions of pain-related activity in the posterior insula during pain stimulation is preceded by stronger activity in pain inhibitory regions such as the PAG, DLPFC and rACC during pain anticipation before the actual pain stimulation is applied. For visualization purposes the images are thresholded at p<0.005. For details see Table 1 F2.

Intriguingly, a positive correlation between the activity in pain-inhibitory areas such as periaqueductal gray (PAG), ACC and DLPFC during the anticipation phase (treated – untreated) and the pain reduction in the posterior insula during the pain phase was observed within the positive treatment history group. This indicates that activity in the descending pain modulatory system during anticipation is associated with more analgesic benefit during pain (Table 1 F2 and Fig. 6).

**Figure 7:**
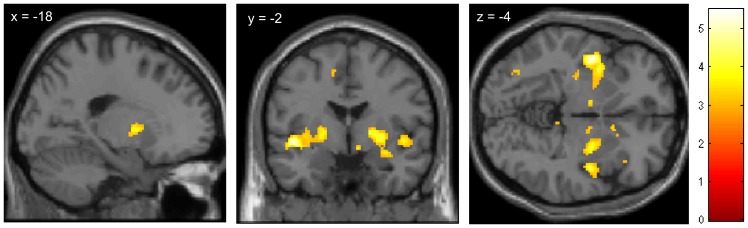
Reduced treatment response in the negative group is associated with increased activity of pain processing regions during the anticipation of pain. Images show BOLD activity (t-scores) during the anticipation phase (t-test: negative correlation of anticipatory activity [treated>untreated site] with the treatment response in the posterior insula for the negative group). For visualization purposes the images are thresholded at p<0.005. For details see Methods and Table 1 F1.

In the negative group we found a negative correlation between activity in pain processing regions such as the insula and the thalamus during the anticipation phase (treated – untreated) and the pain reduction in the posterior insula during pain. This suggests that less analgesic benefit during pain is associated with increased anticipatory responses in pain related areas in this group (Table 1 F1 and Fig. 7).

## Discussion

This study explored how prior experience with one treatment can influence the response to a second subsequently applied different treatment. This was studied in an experimental model of analgesia using an analgesic patch as the first treatment (treatment A) and an analgesic ointment treatment as the second analgesic treatment (treatment B). We found that, in comparison to those subjects with a positive treatment history, participants who experienced no analgesia from the first treatment continued to show a substantially reduced response to a second, different analgesic treatment. These findings therefore indicate that prior treatment experience critically influences the efficacy of a treatment and most importantly, that this influence transfers over time and over therapeutic approach.

Behavioral effects were substantiated by neuroimaging data showing significant activation differences in brain regions coding pain and analgesia between groups. Specifically, the adverse effect of a negative treatment history on analgesia was paralleled by stronger activation in bilateral posterior insular cortices. Activity in this brain region has consistently been shown to correlate with afferent nociceptive input and perceived pain intensity [32] and can therefore be taken as a physiological marker of analgesia. These data provide strong evidence that the differences in analgesia depending on prior treatment history are not the result of report bias, but reflect altered processing of ascending nociceptive input.

Furthermore, we found that the stronger analgesic effect of the second treatment (treatment B) in the positive treatment history group was associated with increased engagement of the right dorsolateral prefrontal cortex (DLPFC) and the anterior cingulate cortex (ACC). This activity not only occurred during painful stimulation but also during the anticipation phase where it scaled with the subsequent reduction of insular activity. The DLPFC and ACC are key structures for top-down modulation of pain through different cognitive factors [33], including placebo and nocebo related modulations of pharmacological effects [34]. Although the multiple ROIs used for small volume correction potentially increased the rate of type 1 error, our data suggest that the descending pain modulatory mechanisms that complement the specific analgesic effect (modelled here by a simple temperature reduction) mediates the effect of treatment history and thereby determines the overall analgesic treatment outcome. This notion is further supported by the observation that activity in the descending pain modulatory system during pain anticipation is positively correlated with the reduction of pain related activity in the posterior insula in the positive treatment history group.

Our neuroimaging data further substantiate findings from previous studies suggesting that specific treatment effects and factors related to the treatment context (i.e. treatment expectancy or treatment experience) ultimately converge at the neurobiological level and can critically modulate the overall treatment outcome [34], [35].

These results significantly extend previous findings by showing that the influence of treatment history transfers over time and over therapeutic approach. The experience with the patch treatment substantially modulated the response to the subsequent ointment treatment, with impaired analgesic efficacy following negative treatment experience and enhanced efficacy after the positive treatment experience.

The results of our study are relevant for several reasons. We provide evidence for the transfer of conditioning effects over different treatment approaches using an experimental longitudinal approach. Because these conditioned effects affect not only responses within the same treatment but also affect subsequent different treatments we refer to these effects as “carry-over effects”.

Data from pharmacological cross-over studies (i.e., drug trials in which subjects receive an active drug and a placebo in a randomized order) have previously suggested the carry-over of treatment effects from placebo to active treatments and vice versa. In a double-blind cross-over study on the effect of an antihypertensive drug, the placebo did not affect the patients' hypertension when administered first [36]. Yet, when placebos were administered after a week-long use of atenolol, they produced a significantly greater antihypertensive response than a ‘no treatment’ condition. Sequence effects of drug efficacy have also been proposed for analgesic treatments of musculoskeletal pain by [37] indicating that not only the placebo medication was more effective following active and effective treatment, but also that the active drug was less effective when it followed an ineffective placebo treatment. Similar sequence effects have recently been reported for the analgesic effect of active compared to sham rTMS for chronic pain [38]. It can, however, not be ruled out that these findings were confounded by residual drug (treatment) effects, the natural course of the underlying disease and expectancy-related effects due to unblinding. Because our study was undertaken in a controlled experimental setting and comprised behavioral and neuroimaging outcome measures it provides strong objective evidence for a carry-over effect in treatment outcome. Importantly, as analgesia was modelled experimentally by using a placebo paradigm, our findings are not related to any drug-specific pharmacological effects and may therefore apply to any treatment.

Our findings on this carry-over effect may have wide implications for medical practice and clinical trial designs. Treatment experiences are ubiquitous in clinical care, particularly in patients suffering from chronic diseases. Carry-over effects might therefore be particularly relevant in chronic conditions where treatments often fail repetitively and negative treatment experiences accumulate along the course of the disease. Our results advocate the careful assessment of treatment history and a strictly systematic treatment approach to avoid negative carry-over effects, as any treatment failure may hamper or even abolish future treatment response. In a similar vein, our results may even challenge common step care approaches in which treatment failure has to precede the prescription of next-in-line interventions [39].

Given that inter-individual differences are a major challenge for medical treatments, we also assessed whether clinically relevant personality traits affect treatment response in general, or specifically with respect to prior treatment experience. We found that higher anxiety levels were associated with lower treatment responses, independent of prior treatment experience. Furthermore, depression scores of our healthy volunteers were negatively correlated with the analgesic treatment response in the positive treatment history group indicating that depression may hamper the positive effects of prior treatment response. This observation highlights that the susceptibility to treatment history effects varies across individuals and indicates that patients with comorbid mood disorders may require particular attention.

Reframing (pharmaco-) therapy as a learning process that impacts therapeutic response over time and over therapy also yields important implications for the design and interpretation of clinical trials [40]. Prior treatment experiences or the participation in drug trials are currently neither systematically assessed nor accounted for in the statistical analysis of clinical trials. This neglect of the influence of treatment history on treatment efficacy might inadvertently contribute to the heterogeneity of responses in clinical trials and thereby reduce assay sensitivity (i.e. the sensitivity for drug placebo differences). Our data strongly suggest that the systematic assessment of the individual treatment history should be an essential part of any clinical trial.

While some implications of our study, such as the assessment of the patients' treatment history, can immediately be applied in the clinic, others require further investigation. In particular, we need to specify the critical time window during which prior effects can affect subsequent outcome, understand how the similarity of treatment approaches impacts the effect, and most importantly, how we can overcome negative and systematically use positive carry-over effects. In our study two locally applied “treatments” (i.e., a patch and an ointment) with presumably different pharmacological profiles were used. According to the conditioning literature carry-over effects are stronger the more similar the conditioned stimuli are in appearance. In support of this notion it has been shown that responses to two subsequent placebo treatments differ depending on treatment similarity [41]. If this holds true for learning effects in drug treatments these findings could motivate the use of different application forms (oral, intravenous, local etc.) after treatment failure in addition to pharmacodynamical considerations. Furthermore, psychological interventions could aid in overcoming the detrimental effects of negative treatment experiences. Additional studies are therefore needed to characterize the role of expectancy induced by the treatment experience.

Taken together, our study provides evidence that treatment history critically determines the response to a subsequent treatment at a behavioral and neurobiological level. Further, we show that the susceptibility for carry-over effects varies across individuals and is associated with trait anxiety and depression. Given the large number of patients with persisting health problems despite various treatment attempts, it seems reasonable to assume that negative treatment experiences and their detrimental effects for subsequent treatment approaches contribute substantially to increased health care costs and importantly, to prolonged suffering of our patients. Even though these experimental findings require replication in larger clinical populations, we feel that awareness of this effect is mandatory for every physician and concerted effort is required to avoid or overcome the negative effects of prior experience on treatment outcome.
